# Development of *In Situ* Gelling and Bio Adhesive 5-Fluorouracil Enema

**DOI:** 10.1371/journal.pone.0071037

**Published:** 2013-08-16

**Authors:** Lu-Lu Wang, Wen-Sheng Zheng, Shao-Hua Chen, Xia-Qin Fang

**Affiliations:** Institute of Materia Medica, Chinese Academy of Medical Sciences & Peking Union Medical College, Beijing City Key Laboratory of Drug Delivery Technology and Novel Formulations, Beijing, China; University of Pécs Medical School, Hungary

## Abstract

In this study, a novel 5-Fluorouracil (5-FU) enema with good bio adhesion and temperature sensitivity was developed using *in situ* gelling technology. The preparation was formulated as a free-flowing liquid before use, while a layer of gel film was quickly formed when administered in the rectum, with a large contact surface area. It also demonstrated good biocompatibility, appropriate gel strength and bio adhesive force with excellent adhesion to rectal mucosa and prolonged action time, allowing more effective drug absorption and diffusion to surrounding tissues. Poloxamer 407 and poloxamer 188 were applied to adjust the gelling temperature. With the addition of carbopol and polycarbophil (bio adhesive substances), the solubility of 5-FU and gel strength increased, the temperature of gelation and the surface area of drug contact on mucous epithelium decreased. Decreased adhesive force between the preparation and the mucous membrane of the rectum was demonstrated with improving carbopol and polycarbophil’s concentration. In vitro release demonstrated that 5-FU *in situ* gelling enema with different bases had a rapid and almost complete drug release. We used an optimized formulation of P407/P188/polycarbophil/5-FU (17/2.5/0.2/1.0) for animal experiments. The result showed that the drug evenly covered the surface of the rectum and there was no leakage in 6 hours. The *in situ* gelling enema showed significantly higher rectal tissue levels of 5-FU compared with suppository and intravenous administration, indicating that 5-FU could be well absorbed due to the enlarged releasing area, longer retention time and larger amount of dissolved active ingredients. Systemically, 5-FU levels in the enema group were similar to those in the suppository group and significantly lower than the intravenous group. The enema was not associated with morphological damage to rectal tissue. These results suggest that the bio adhesive and *in situ* gelling enema could be a more effective rectal delivery system of 5-FU.

## Introduction

5-Fluorouracil (5-FU) is an antimetabolic anticarcinoma agent that exerts its effect through competitive inhibition of thymidylate synthetase, resulting in thymine deficiency and impaired DNA synthesis [1]. It is also taken up as an RNA precursor leading to defective RNA synthesis [2]. For several decades, 5-FU has been widely used for the treatment of malignancies arising from breast, gastrointestinal tract, head and neck regions [3]. 5-FU is the main chemotherapeutic agent used for the treatment of colorectal cancer [4]–[6]. It is given intravenously via bolus or continuous infusion in doses ranging from 250 to 1,000 mg/m^2^/day [7]–[10]. 5-FU toxicity is predominantly dependent on the mean serum concentration and typical adverse events include leukopenia, diarrhea, stomatitis, and weight loss [7]–[10].

Regional administration of 5-FU has been proposed as an alternative method to improve the toxicity profile [11], [12]. In Japanese clinical studies, 5-FU administered via suppository was reported to be associated with less systemic toxicity, while permitting direct topical contact with rectal cancer [13], [14]. These initial reports prompted interest in the local delivery of 5-FU for the treatment of colorectal cancer. Numerous studies showed that suppository administration of 5-FU appear to be efficient and show similar pharmacokinetics to intravenous delivery, but associated with fewer systemic side effects and higher rectal tissue drug concentrations [15]. Some studies in animal models also demonstrated that after rectal administration in the form of an emulsion, 5-FU not only remained in the rectal mucosa for longer, but also accumulated in regional lymph nodes at high concentrations for long periods, suggesting that 5-FU emulsion therapy may have a role to play in the treatment of lymph node metastasis associated with rectal cancer [16], [17]. Use of conventional solid type suppositories is often accompanied by discomfort, which leads to poor compliance. Furthermore, 5-FU suppositories often cause anal pain, tenesmus, anal bleeding and reddened mucosa with histological changes compatible with acute colitis [13], [18]. Liquid forms often cause anal leakage leading to inadequate dosing.

The development of a new thermosensitive liquid suppository has prompted investigation of many drugs with this new method of delivery: indomethacin [19], [20], acetaminophen [21]–[23], diclofenac sodium [24], [25], propranolo [26], quinine [27], insulin [28], libuprofen [29], carbamazepine [30], ondansetron [31], diltiazem hydrochloride [32], etodolac [33], nimesulide [34], curcuminoids [35], short-chain fatty acids [36], and tramadol hydrochloride [37]. Overall, administration with the newer liquid suppositories leads to improved patient compliance and increased drug bioavailability. While many previous studies have focused on modulating the gelation temperature and bio adhesive effects of polymer solutions, there is a lack of knowledge on the extent of surface area contact with liquid gels and mucous epithelium of rectum. This factor is crucial in designing desirable dosage formulations that will influence bio adhesive force, release properties, absorption rate and bioavailability of the drugs. In addition, liquid suppository cannot ameliorate rectal irritation and insufficient absorption associated with poor water soluble drugs.

Taking the above factors into consideration, we developed a thermosensitive and bio adhesive rectal enema for 5-FU administration. The physicochemical characteristics such as gelation temperature, surface contact area, bio adhesive force, gel strength and in vitro release properties were evaluated. The optimized formulation was prepared and infused into rabbit rectum to test the in vivo spread and retention properties. The 5-FU concentration in rat’s rectal tissue and systemic blood after the administration of 5-FU *in situ* gelling enema (5-FU-E), 5-FU suppository (5-FU-S) and 5-FU intravenous (5-FU-IV) was investigated, then, rectal tissue irritation of the 5-FU-E was assessed. The relationships between the surface contact area of 5-FU and properties such as bio adhesive force, in vitro release, absorption and rectal irritation were also documented. Our results indicated that the 5-FU *in situ* gelling enema could be more efficient for the treatment of rectal cancer and may have an improved tolerability profile.

## Materials and Methods

These studies were carried out in strict accordance with the recommendations in the Guide for the Care and Use of Laboratory Animals of the National Institutes of Health. The protocol was approved by the Committee on the Ethics of Animal Experiments of the Chinese Academy of Medical Sciences & Peking Union Medical College. The approval specifically covered this portion of the work with rabbits and rats. All surgery was performed under sodium pentobarbital anesthesia, and all efforts were made to minimize suffering.

### Materials

Poloxamers were gifted from BASF (Ludwigshafen, Germany). Carbopol (974P NF) and polycarbophil (Noveon AA-1) were gifted from Lubrizol Corporation (Cleveland, America) and 5-Fluorouracil was purchased from Xing galaxy chemical company (Hubei China). All other reagents were of analytical grade. Water was distilled before use.

### Solubility of 5-FU in Carbopol and Polycarbophil

Excess 5-FU (2 mg) was added to a glass bottle containing 10 mL of deionized water with various amounts of carbopol or polycarbophil. All measurements were carried out in triplicate. Solutions were shaken at 25°C for 120 hours. After equilibrium was reached, the contents of each bottle were filtered through a 0.45 µm micropore membrane and the remaining filtrate was collected for analysis of 5-FU.

### Preparation of *in situ* Gelling Enemas

The cold method was adopted for preparation of *in situ* gelling enemas [19], [20], [24], [26]. Formulations were prepared by weight and various amounts of excipients except poloxamers were completely dispersed in distilled water with continuous agitation at room temperature for solution A. P407 and P188 were added slowly into cold water and the liquid was placed at 4°C until clear solution B was obtained. An appropriate amount of solution A and B were mixed to obtain solutions containing 1.0% 5-FU and carbopol or polycarbophil with a concentration range of 0.2%–0.8%.

### Determination of 5-FU Contents in Enema

Each enema (0.5 g) was dissolved in 250 mL distilled water and then filtered. The solutions were analyzed using high performance liquid chromatography (System conditions: Waters high performance liquid chromatography including 515 single pump, 2487 detector, 717 autosampler, chromstation work station; column: a MN C_18_ column (150 mm×4.6 mm, 5 µm) from MACHEREY-NAGEL GmbH& Co. KG; mobile phase: acetonitrile–water (95∶5, pH 3.5); flow rate: 1 mL·min^−1^; room temperature; detection wavelength: 267 nm; injection volume: 20 µL). The standard curve A = 63.489X+12.454 (r = 0.9990) was plotted with the peak area of 5-FU as ordinate against its concentration. The linear range was 0.1–20 µg·mL^−1^ and recovery rate was 99.1%. The RSD (Relative Standard Deviation) for repetition detected at 0, 4, 12 and 24 h were 3.1%. The RSD for repetition of six times were 1.9% with peak area as index and 3.1% by detecting 5-FU peak area.

### Measurement of Gelation Temperature

The gelation temperature was measured using procedures reported by Choi et al [19], Ryu et al [24], and Yun et al [26]. A 20 mL transparent beaker containing a magneton and 10 g of liquid *in situ* gelling enema was placed in a low-temperature thermostat water bath. A digital thermal element was immersed into the solution. Liquid enema was then heated at a constant rate with continuous stirring. When the magneton stopped moving due to the gelation, the temperature displayed on the thermistor was identified as a gelation temperature.

### Measurement of Gel Strength

CT3 Texture Analyzer (Brookfield Engineering Laboratories, Middleboro, MA, USA) was used to test the gel strength. Liquid *in situ* gelling enema (40 g) was placed in a 100 mL beaker and gelled at 36.5°C. The gel strength was determined by the force (g) required to move the chapiter 7 mm down through the gel.

### Measurement of Surface contact Area

To measure the surface contact area of *in situ* gelling enema, male New Zealand rabbits weighing 2500 g were sacrificed by anesthesia and bleeding. The colorectal tract with the mesenteric arcade attached, was excised and placed in physiological salt solution (PSS). The connective tissue was dissected and cleaned then secured with the mucosal side out on a glass plate, raised at a 10 degree slope and stored in an oven at 36.5°C. 1 mL of liquid enema was administered on the surface of the rabbit rectum, allowed to spread freely until the formation of a gel film occurred. The area of gel surface contact area was calculated.

### Determination of Bio Adhesive Force

**Figure 1:**
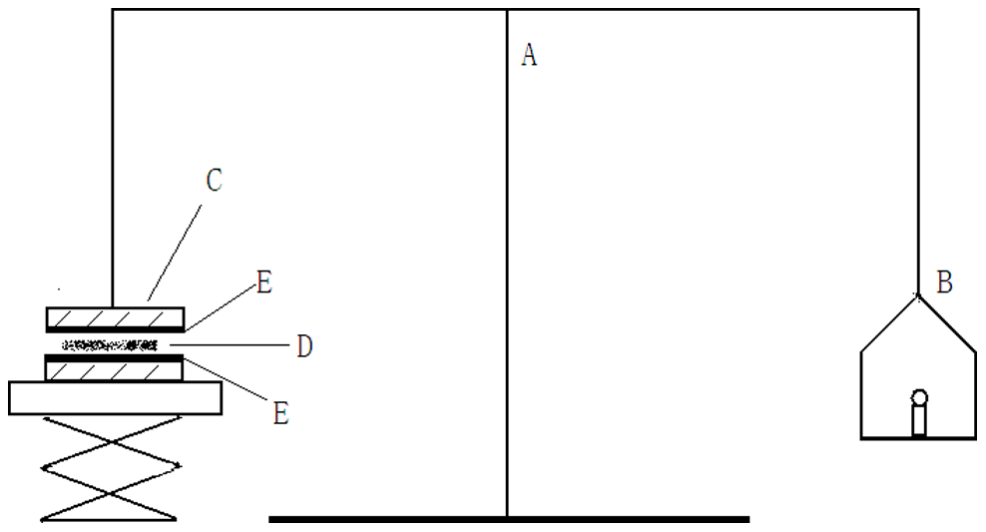
Bio adhesive force-measuring device: (A) modified balance; (B) weights; (C) metal plate; (D) *in situ* gelling enema; (E) rectal tissue.

Referencing the previously reported methods [21], [24], [26], the bio adhesive force of *in situ* gelling enema was determined by using the modified measuring device shown in Figure 1. In brief, male New Zealand rabbits weighing 2500 g were sacrificed by anesthesia and bleeding. The colorectal tract with the mesenteric arcade attached, was excised and placed in physiological salt solution (PSS). The connective tissue was dissected and cleaned, then secured with the mucosal side out, onto each metal plate (C) using a rubber band. The experiments were conducted under 36.5°C. One metal plate with a section of tissue (E) was connected to the balance and another plate was placed on a height-adjustable pan. 1 mL of liquid *in situ* gelling enema was placed on the surface of the rectum and spread freely. The height of another plate was adjusted so that the gel could adhere to the tissue of the upper plate and remain in place for five minutes. The weights (B) were kept raised until the two plates were separated. Bio adhesive force was determined from the minimal weights that separated the two plates.

### Release Test

The *in vitro* release of 5-FU was determined by dynamic dialysis. One gram of the enema was transferred to prepared dialysis channels, then placed in a conical flask filled with 100 mL deionized water solution and magnetically stirred at 37°C at 100–150 r⋅min^−1^. 2 mL of each sample was taken at 5, 10, 20, 30, 45, 60, 90 and 120 minutes respectively and the same volume of deionized water was added simultaneously. The concentration of dialyzed drug was determined by high performance liquid chromatography (mentioned before) for calculation of cumulative ability to dialyze. The absorption of drug was not altered by materials at 265 nm. To understand the mechanism of *in vitro* release, we analyzed the data with DD Solver (1.0) software.

### Identification and Retention of Liquid Enema *in vivo*

The methods of Choi et al [19], [20], Ryu et al [24], were adopted for this determination. The procedural details were as follows: Male, New Zealand rabbits weighing 2500 g were fasted for 24–36 hours prior to the experiment and allowed free access to water. Following sodium pentobarbital anesthesia, liquid enema P407/P188/polycarbophil/5-FU (17%/2.5%/0.2%/1.0%) and 5-FU (1.0%) water solution with 0.1% blue lake was administered at a dose of 2 mL⋅kg^−1^ into the rectum 1 cm above the anus using a stomach probe needle. At 5 minutes and 6 hours after administration, the rabbits were sacrificed by stunning and bleeding. The rectum was sectioned and the identification and retention of *in situ* gelling enema in the rectum was documented. Five rabbits were used for each measurement.

### Concentration of 5-FU in Rectal Tissues and Systemic Blood

#### Preparation of 5-FU suppository

Wite pol was heated to 55°C, then 5-FU was slowly added to the solution with continuous agitation. The resulting solution was placed into the suppository mould and cooled down to 25°C. Each 0.1 mL suppository contained 5 mg 5-FU.

#### Drug treatment

Male Spragure-Dawley rats (∼300 g) were used for this study. Sixty-two rats were divided into three groups (22, 20, 20) and deprived of food for 24 hours prior to drug administration. 5-FU enemas (5-FU-E) were administered at a dose of 2 mL· kg^−1^ (20 mg· kg^−1^ as 5-FU) into the rectum using a stomach sonde needle fitted to a plastic syringe. For comparison of bioavailability, 5-FU suppositories (5-FU-S) were administered at a dose of 0.4 mL kg^−1^ (20 mg· kg^−1^ as 5-FU) into the rectum and an aqueous solution of 5-FU (4%, w/v) was also administered intravenously at the same dose (1.0 mL·kg^−1^ as volume and 20 mg kg^−1^ as 5-FU) to rats according to standard methods. The dose was equal to three times the human dose (400 mg· 60 kg^−1^) [16], [17], [38].

#### Animal experiments

The method of Galandiuk et al [16] was adopted for this investigation. At thirty minutes, 1, 3, 6 and 12 hours after drug administration, five animals from each group were anesthetized. Cardiac puncture was performed to collect systemic blood and to sacrifice by exsanguination. Rectal tissues were harvested to determine 5-FU tissue concentrations. Serum and tissue samples were stored at −70°C until analysis.

#### Pretreatment of tissue sample

The obtained tissue sample was mashed and ground into a homogenate by adding 2 mL of purified water three times. After standing for 0.5 hours, the precipitate was removed by centrifugation, washed and filtered with 0.4 mL purified water. 1 mL of 0.5 mol·L^−1^ ammonium sulfate addition to combined filtrate led to proteins precipitation. Following homogenization, 1.5 mL of ethyl acetate was added in three equal portions (0.5 mL, 0.5 mL, 0.5 mL). Vortex mixer was applied for drug extraction for 0.5 minutes after each ethyl acetate addition. Subsequently, the ethyl acetate layer was collected and centrifuged at 4,000 r· min^−1^ for 5 minutes. 1 mL of supernatant was measured accurately, removed to a peaked bottom tube and dried by nitrogen gas in water bath at 40°C. Then the residue was dissolved in 100 µL of mobile phase of which 20 µL was measured accurately for determination.

#### Preparation of plasma samples

Protein precipitation was carried out by adding 1 mL of 0.5 mol· L^−1^ ammonium sulfate to 0.5mL of plasma placed in a stoppered centrifuge tube. After homogenization, 1.5 mL of ethyl acetate was added in three equal portions (0.5 mL, 0.5 mL, 0.5 mL). Drug extraction was carried out on a vortex mixer for 0.5 minutes after each ethyl acetate addition. Then the ethyl acetate layer was collected and centrifuged at 4,000 r· min^−1^ for 5 minutes. 1 mL of supernatant (ethyl acetate solution) was measured accurately, removed to a peaked bottom tube and dried by nitrogen gas in water bath at 40°C. Subsequently, the residue was dissolved in 100 µL of mobile phase of which 20 µL was measured accurately for determination.

#### Determination of 5-FU

The determination of 5-FU was carried out by high performance liquid chromatography (mentioned before). The standard curve Y = 102.317X+21.436 (r = 0.9991) was plotted with the peak area of 5-FU as ordinate against its concentration as abscissa. The linear range was 0.5–50 µg· mL^−1^ and the recovery rate was 92.3%. The RSD for repetition detected at 0, 4, 12 and 24 h was 3.1%. The RSD for repetition of six times was 4.5% with peak area as index. The minimum detectable amount was 5 ng.

### Statistics

Data were analyzed by Student’s t-test for significant at a level of 0.05.

### Rectal Irritation

Immediately after the final animal sampling from concentrations of 5-FU in rectal tissue and systemic blood test, two animals from the 5-FU-E group were chosen. The rectum was isolated, washed with saline solution, fixed in 10% neutral carbonate-buffered formaldehyde, embedded in paraffin and cut into slices. The slices were stained with hematoxylin-eosin and observed by light microscopy for morphology [24].

## Results

### Solubility of 5-FU in Carbopol® and Polycarbophil®

**Figure 2:**
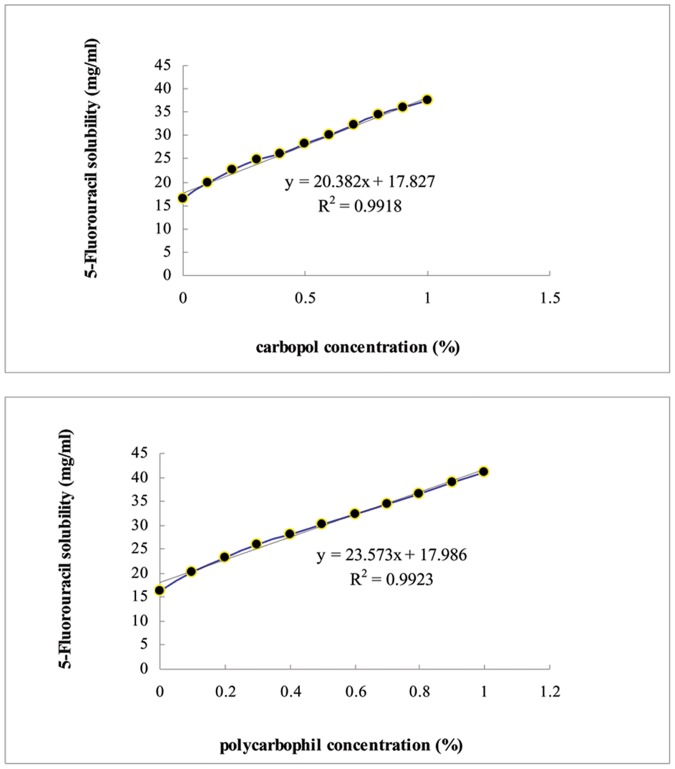
Effects of carbopol and polycarbophil on the aqueous solubility of 5-FU. Each value represents the mean ± SD (n = 3).

To determine solubility enhancement of carbopol and polycarbophil for 5-FU, the phase solubility diagram of 5-FU with carbopol and polycarbophil is shown in Figure 2. Both carbopol and polycarbophil greatly improved the aqueous solubility of 5-FU and was proportional to the concentration of carbopol and polycarbophil. Polycarbophil had greater functionality in soluble solutions than carbopol.

### Gelatin Temperatures of *in situ* Gelling Enemas

**Figure 3:**
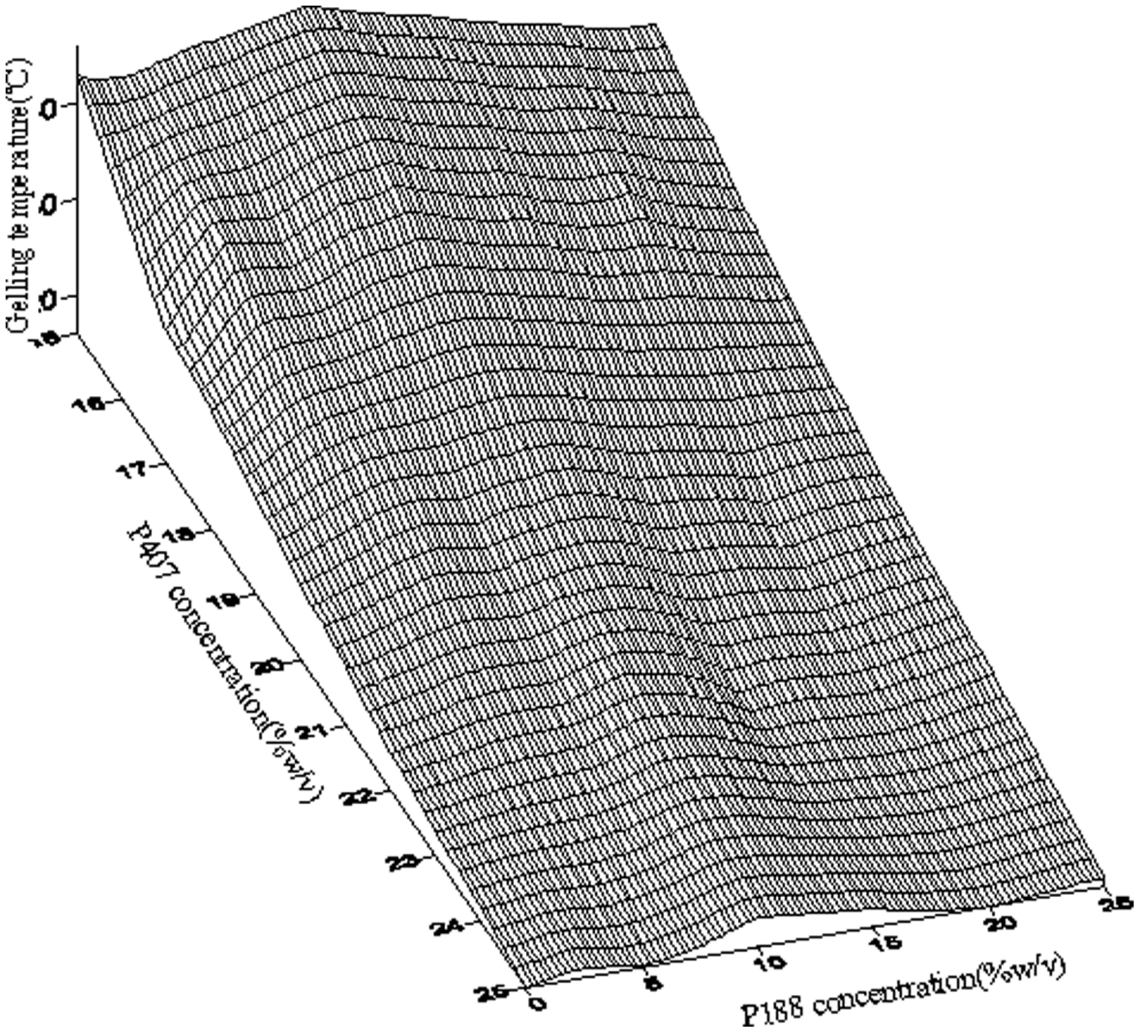
Effect of P407 and P188 concentrations on the gelling temperature (n = 3).

**Figure 4:**
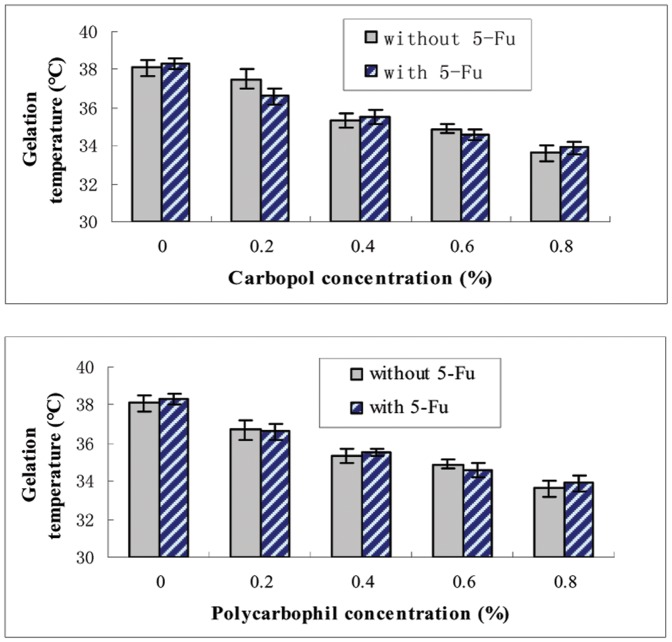
Effect of contents of 5-FU, carbopol and polycarbophil on the gelation temperature. The gelation temperature of poloxamer was measured in various concentrations of carbopol and polycarbophil. Poloxamer compositions of P407/P188 were 17/2.5 (w/v). The concentration of 5-Fluorouracil (5-FU) was 1.0%, each point represents the average of three separate experiments.

As showed in Table 1 and Figure 3, gelation temperature of formulations decreased relative to the concentration of P407 and P188 was not as influential as P407. Gradual addition of P188 with constant P407 concentration resulted in increasing gelling temperature levels to a maximum when P188 concentration was 10%, followed by reducing levels. We selected 17% P407 and 2.5% P188 solutions for further investigation. It was reported that bio adhesive polymers and active ingredients influenced the temperature of gelation [19], [25], [26], [29], [30]. The temperature of various formulations with different concentration of carbopol, polycarbophil and 5-FU were tested. The bio adhesive polymer had a concentration dependent T sol-gel lowering effect (Figure 4). However, 5-FU did not influence the gelation temperature. 1% 5-FU-E with P407/P188 (17/2.5) and 0.4–0.8% carbopol or 0.2–0.8% polycarbophile had suitable gelation temperatures.

**Table 1:** Effect of P407 and P188 concentrations on the gelling temperature (n = 3).

Gelling	temperature(°C)		P407	(%, w/v)		
		15	17	20	22	25
	2.5	42.1±0.4	38.1±0.4	26.3±0.4	21.3±0.2	17.3±0.2
	5	44.1±0.2	37.5±0.1	28.5±0.1	22.2±0.1	16.5±0.3
P188	7.5	45.3±0.3	40.6±0.2	27.1±0.2	22.5±0.1	17.6±0.3
(%, w/v)	10	46.4±0.5	41.2±0.2	28.7±0.3	24.6±0.2	19.3±0.5
	15	43.2±0.2	38.3±0.4	26.6±0.1	20.9±0.3	18.3±0.1
	20	40.5±0.1	36.8±0.3	24.3±0.2	20.4±0.4	16.2±0.1
	25	38.3±0.1	32.5±0.2	25.5±0.5	22.2±0.3	17.5±0.2

### Gel Strength of *in situ* Gelling Enemas

**Figure 5:**
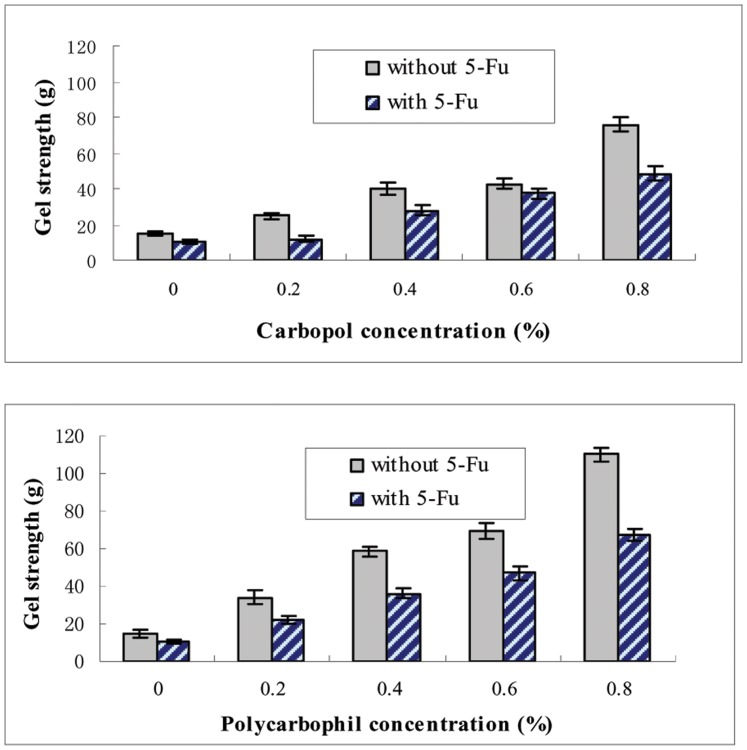
Effect of contents of 5-FU, carbopol and polycarbophil on the gel strength. The strength of gelled poloxamer was measured in varying concentrations of carbopol and polycarbophil. Poloxamer’s compositions of P407/P188 were 17/2.5 (w/v), the concentration of 5-FU was 1.0%. Gel strength was measured by the time that the apparatus itself (35 g) without any weights move down the gel. Each point represents the average of three separate experiments.

The strength of gelled enema was measured in varying concentrations of carbopol (A) and polycarbophil (B). Poloxamer compositions of P407/P188 were 17/2.5 and the concentration of 5-FU was 1.0%. Gel strength was measured by the force it took to move the chapiter 7 mm down through the gel and each point represented the average of three separate experiments. 5-FU reduced the strength of poloxamer gels and also affected the strength of the Poloxamer gels containing bio adhesive polymers (Figure 5). Carbopol and polycarbophil greatly enhanced gel strength, carbopol and polycarbophil enhanced gel strength to a similar extent in P407/P188 (17/2.5) with or without 5-FU. Carbopol and polycarbophil reinforced the gel strength in proportion to their concentrations. The range of gel strength suitable for *in situ* gelling enema was investigated by insertion into the anus of rabbit and observing for leakage (gel strength higher than 20g does not causes anal leakage). 1% 5-FU-E with P407/P188 (17/2.5), carbopol higher than 0.4% or polycarbophil higher than 0.2% had enough gel strength to be retained in the rectum.

### Surface Contact Area of *in situ* Gelling Enemas

**Figure 6:**
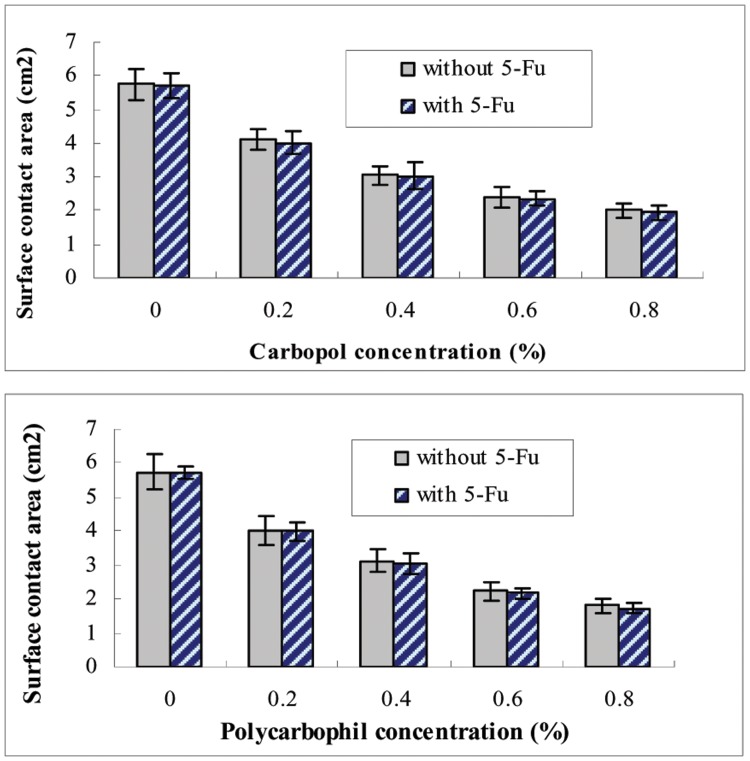
Effect of contents of 5-FU, carbopol and polycarbophil on the surface contact area. The surface contact area of poloxamer was measured in various concentrations of carbopol and polycarbophil. Poloxamer’s compositions of P407/P188 were 17/2.5. The concentration of 5-FU was 1.0%. Each point represents the average of three separate experiments.

Carbopol and polycarbophil decreased the area of spread of gelled poloxamer (Figure 6). The decrease of surface contact area indicated relatively good linearity over bio adhesive polymer concentrations. It is noteworthy that polycarbophil showed a greater extent of reducing the area of spread compared with carbopol and 5-FU had no influence on the surface contact area.

### Bio Adhesive Force of *in situ* Gelling Enemas

**Figure 7:**
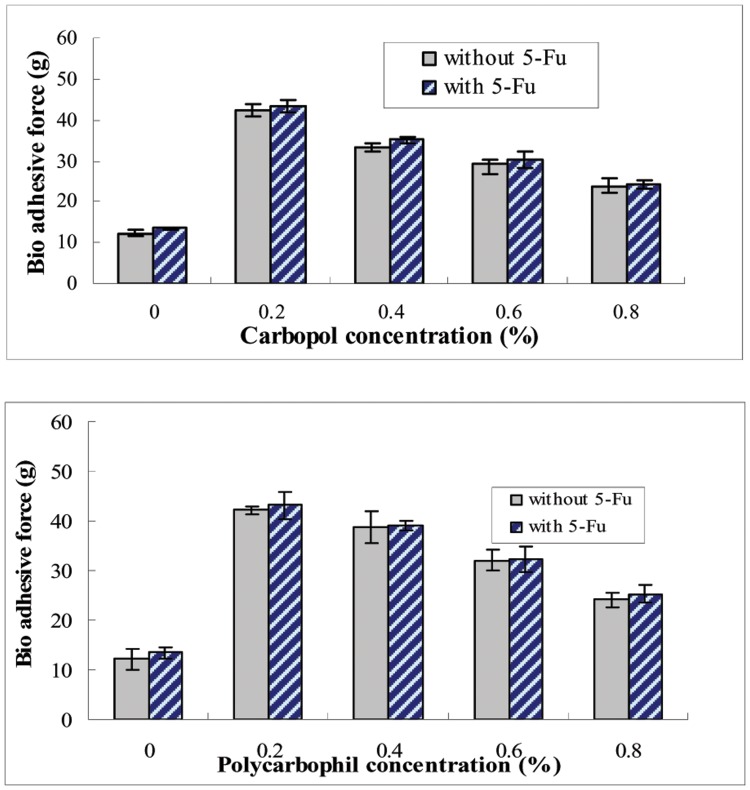
Effect of contents of 5-FU, carbopol and polycarbophil on the bio adhesive force. The bio adhesive force of poloxamer was measured in various concentrations of carbopol and polycarbophil. Poloxamer compositions of P407/P188 were 17/2.5. The concentration of 5-FU was 1.0%. Each point represents the average of three separate experiments.

Liquid gel was added to the surface of rectal tissue, allowed to spread and form a solid gel film, then,tested for detachment stress. 5-FU decreased the bio adhesive force of the gels containing carbopol and polycarbophil (Figure 7). Contrary to results from other studies [39]–[48], the bio adhesive force of the gels decreased with increase of carbopol or polycarbophil’s concentration and showed a positive correlation with the surface contact area of the formulation. However, our experiments showed that the bio adhesive force was not proportional with the surface contact area of the gel (data not shown). This indicates that the bio adhesive characteristic of the formulation was influenced not only by bio adhesive ingredients characteristics, but also by the surface contact area with mucosal tissue. The gel’s surface contact area contributed more to the bio adhesive force.

### *In vitro* Release

**Figure 8:**
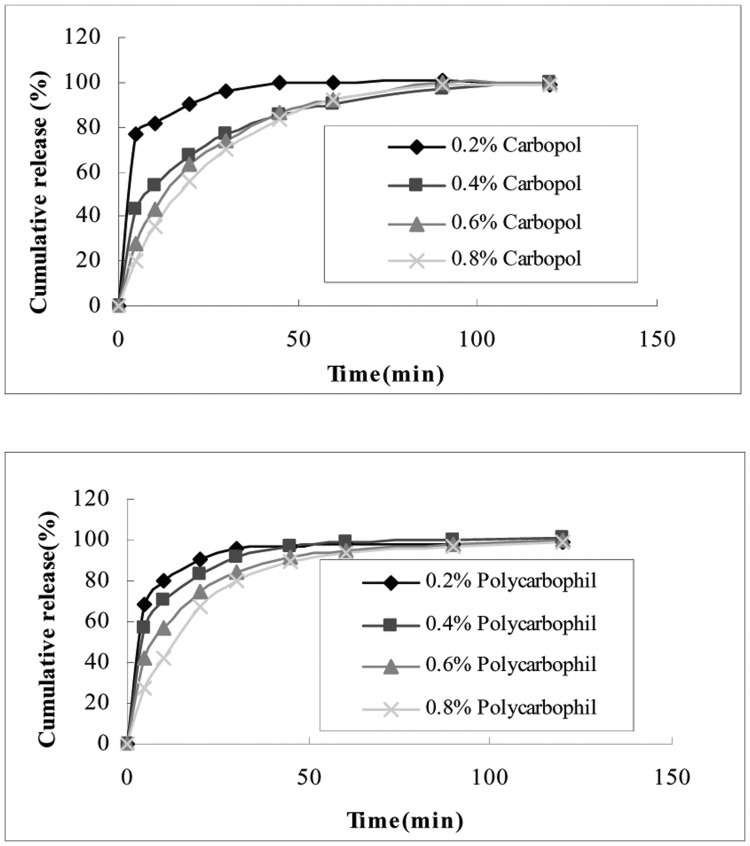
The effect of carbopol and polycarbophil’s concentration on the release of 5-FU. *In situ* gelling enemas were composed of 0.2–0.8% carbopol or polycarbophil. Poloxamer compositions of P407/P188 were 17/2.5. The concentration of 5-FU was 1.0%. Each point represents the average of three separate experiments.

The release test was performed with formulations consisting of a constant amount of P407 (17%), P188 (2.5%) and variable amounts of carbopol (0.2–0.8%) and polycarbophil (0.2–0.8%). The release area was determined according to the surface contact area and calculated from the surface contact area studies. Additionally, the accumulative release percentage of 5-FU was calculated. 5-FU-E, with different bases, showed rapid and almost complete drug release character with 90% of 5-FU released within one hour (Figure 8). Although the release rates of 5-FU from enemas tended to decrease as the concentrations of carbopol and polycarbophil increased, it did not show sustained release properties as described in other studies. [19] [24] To understand the release mechanisms of 5-FU from *in situ* gelling enemas, data was analyzed with DD Solver (1.0) software. The results indicated that 5-FU might be released from the enema by the Makoid-Banakar model with carbopol and Weibull model with polycarbophil.

### Retention of Liquid Enema *in vivo*

**Figure 9:**
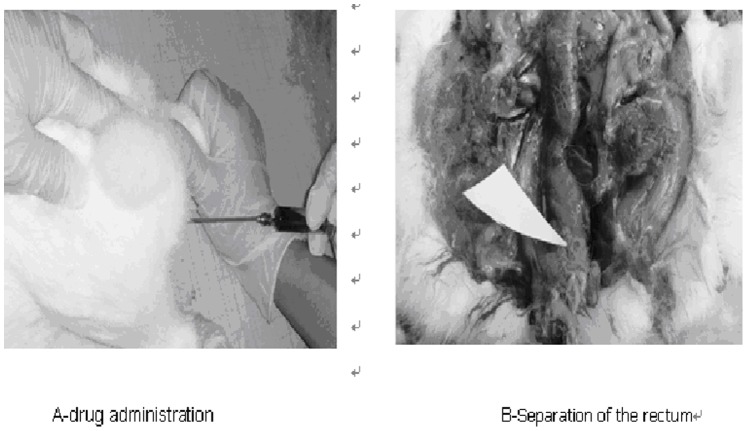
Drug administration and separation of the rectum.

**Figure 10:**
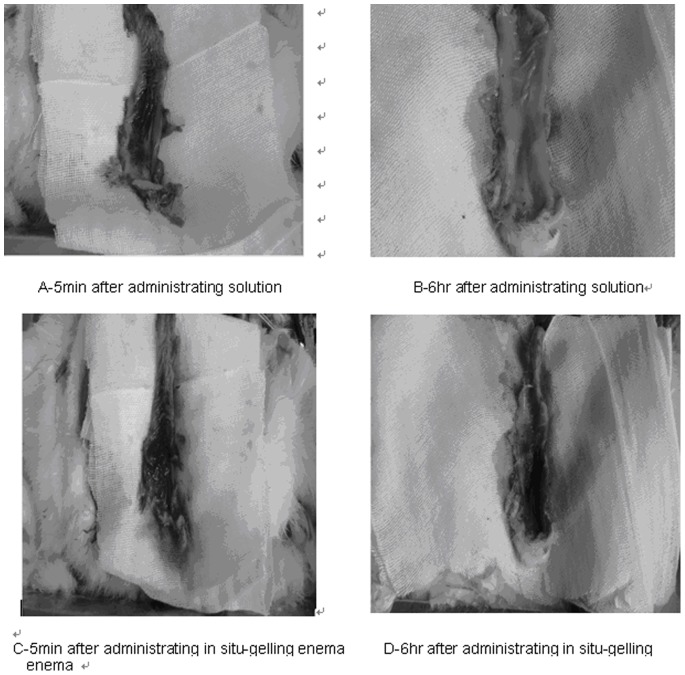
Drug retention in the rectum.

From the results of previous experiments, *in situ* gelling enema composed of P407/P188/polycarbophil/5-FU (17%/2.5%/0.2%/1.0%) with a suitable gelation temperature, gel strength, larger surface contact area and greater bio adhesive force, was administered into rats and retention in the rectum was observed (Figures 9 and 10). 5-FU (1%) water solution was applied as a reference. Five minutes after administration, the blue color of the *in situ* gelling enema and the water solution were clearly shown in the rectum. The enema covered the superior surface of rectum and formed a gel film. Six hours following administration, the position and shape of *in situ* gelling enema in the rectum did not greatly change with time, yet, the water solution faded. This suggested that the bio adhesive force of *in situ* gelling enema was strong enough to hold the gelled enema in the rectum of rabbits for at least 6 hours.

### Concentration of 5-FU in Rectal Tissues and Systemic Blood

**Figure 11:**
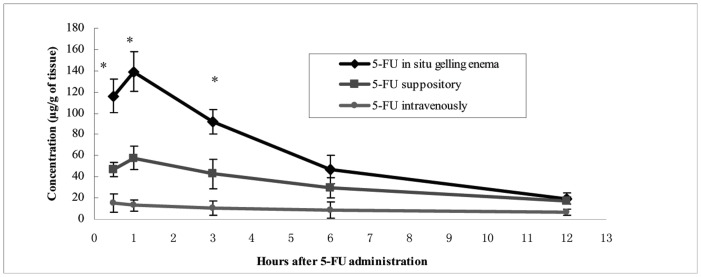
5-FU concentrations in rectal tissue over time after administration of 20 mg·kg^−1^ 5-FU intravenous, suppository or *in situ* gelling enema. *p<0.05.

**Figure 12:**
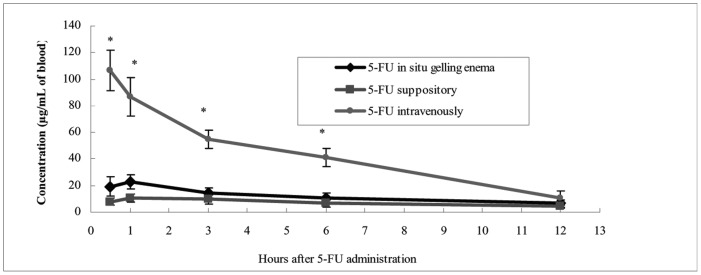
5-FU concentrations in systemic blood over time after administration of 20 mg·kg^−**1**^ 5-FU intravenous, suppository or *in situ* gelling enema. *p<0.05.

5-FU concentrations in rectal tissue were significantly higher in 5-FU-E animals during the first three hours compared with the 5-FU-S and 5-FU-IV groups (p<0.05) (Figure 11). 5-FU concentrations in systemic blood in 5-FU-E and 5-FU-S groups were similar and both were significantly less than that of 5-FU-IV group (p<0.05) (Figure 12).

### Rectal Irritation

**Figure 13:**
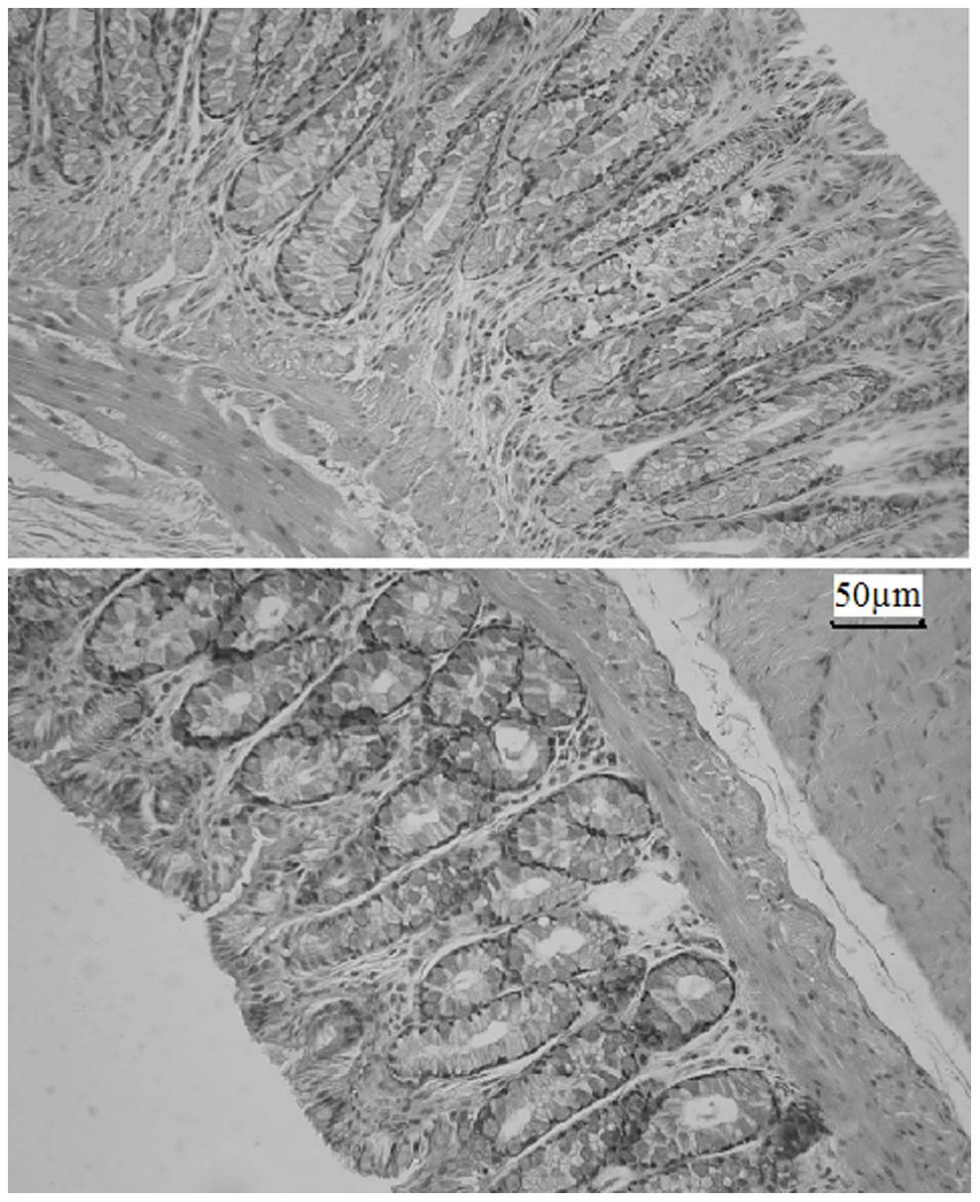
The morphology of rectal tissues.

The morphology of rectal tissues showed in Figure 13, indicated that 5-FU-E did not irritate or damage rectal tissues during the 12 hour observation period.

## Discussion

Carbopol® polymer and Noveon® polycarbpohil are high molecular weight, cross-linked homopolymers and copolymers of acrylic acid. They provide excellent bio adhesive properties and have been used for the formulation of buccal cavity, vaginal, nasal ophthalmic and rectal bio adhesive products. In this study, Carbopol® polymer and Noveon® polycarbpohil increased the solubility of 5-FU by providing high hydrogen ion content. Polycarbophil increased 5-FU’s solubility at a greater rate than carbopol. This could be explained by the high molecular weight of polycarbophil, an acrylic acid polymer cross-linking with divinyl glycol and the hydroxyl terminal can form hydrogen bonding with 5-FU.

As previously describe by Dr. Han-Gon Choi [19], [20], gelation temperature is defined as the temperature at which the liquid phase makes a transition to gel. A gelation temperature range suitable for liquid enema is typically 30–36°C. As bases of liquid enema, poloxamer 407 (P407) and poloxamer188 (P188) were selected because of their therm-sensitive gelling properties, low toxicity, less skin irritation, excellent water-solubility and compatibility with other chemicals.

Bio adhesion is defined as the attachment of macromolecules to biological tissue [39]–[43]. Concepts of bio adhesion have received great interest in pharmaceutical science in recent years, for its ability to prolong the residence time and adherence to mucosal tissue. Research has focused on explaining mechanisms of bio adhesion. Bio adhesive properties can be explained by surface energy thermodynamics and interpenetration/diffusion [44]–[50]. Firstly, the bio adhesive materials must make contact and interpenetrate with mucosal tissue, therefore, the contact area is an important property of bio adhesives. Our study revealed that, contrary to the results of others, the bio adhesive force of the gels decreased with improvement of carbopol or polycarbophil’s concentrations and was positively correlated with the spread area of the formulation. We speculate that with the decreasing concentrations of carbopol or polycarbophil’s, formulations could posses a large contact area with rectal mucous epithelium with increased interfacial bond formation, creating a stronger bio adhesive force. The bio adhesive character of the formulation was influenced not only by bio adhesive ingredient character, but also by the area of contact with mucosal tissue.

The release properties of a few drugs from P407 gels have been reported [19], [21]–[24], [26], [51]–[57]. Most of these studies examined diffusion of the drug from the gel in either aqueous or non-aqueous media and with or without an intervening membrane. These models showed that drug release follows the Higuchi square root law and the diffusion coefficient of a drug in the gel, decreases with increasing P407 content and bio adhesive material content, consistent with a consequent increase in bulk viscosity and gel rigidity. These models permit a good understanding of drug release in forms used for oral, nasal, vagina and rectal sites. However, in our study, when the *in situ* gelling enema was applied to the rectum, a thin film was successfully formed and covered a large area of the rectal mucosa epithelium, thus, the drug releasing area should be considered an important component of its mechanism of action. 5-FU-E that we designed with different bases, showed rapid and almost complete drug release characteristics, with 90% of 5-FU released within one hour.

It is interesting that high rectal concentrations persisted for up to three hours after 5-FU-E administration, whereas, conventional suppositories remained at lower rectal concentrations. This could be explained by 5-FU-E having a large releasing area, longer retention time and contained more dissolved active ingredients than 5-FU-S. We found that the concentration of 5-FU was low in systemic blood, which could manifest as improved therapeutic efficacy and a reduced adverse event profile.

Morphological studies showed that 5-FU-E did not irritate or damage rectal tissue during a 12 hour period, probably due to their greater area of contact and relatively low drug concentration on the rectal mucous membrane.

### Conclusion

The *in situ* gelling enema we designed, can flow freely at room temperature and converts into a thin film lining the rectal mucosa after application. The enema becomes evenly spread and adheres strongly. This spread is an advantage of this dosage-form and the large spread area may improve the drug release, absorption and tolerability.
